# Body Composition and Basal Metabolic Rate in Women with Type 2 Diabetes Mellitus

**DOI:** 10.1155/2014/574057

**Published:** 2014-11-10

**Authors:** Marina de Figueiredo Ferreira, Filipe Detrano, Gabriela Morgado de Oliveira Coelho, Maria Elisa Barros, Regina Serrão Lanzillotti, José Firmino Nogueira Neto, Emilson Souza Portella, Haydée Serrão Lanzillotti, Eliane de Abreu Soares

**Affiliations:** ^1^Nutrition and Health of Nutrition Institute, State University of Rio de Janeiro, St. São Francisco Xavier, No. 524, Block D, 12° Floor, Maracanã, 20559-900 Rio de Janeiro, RJ, Brazil; ^2^Nutrition Institute, Federal University of Rio de Janeiro, Aloízio Avenue, No. 50, Granja dos Cavaleiros, 27930-560 Macaé, RJ, Brazil; ^3^Nutrition Department, Estácio de Sá University, St. Bispo, No. 83, 20261-063 Rio de Janeiro, RJ, Brazil; ^4^Nutrition Department, Arthur Sá Earp Neto College, Barão do Rio Branco Avenue, No. 1003, 25680-120 Petrópolis, RJ, Brazil; ^5^Mathematics and Statistics Institute, State University of Rio de Janeiro, St. São Francisco Xavier, No. 524, Block B, 6° Floor, Maracanã, 20559-900 Rio de Janeiro, Brazil; ^6^Lipid Laboratory, Faculty of Medical Science, State University of Rio de Janeiro, Avenida Marechal Rondon, No. 381, 20950-003 Rio de Janeiro, RJ, Brazil

## Abstract

*Objective.* The aim of this study was to determine which of the seven selected equations used to predict basal metabolic rate most accurately estimated the measured basal metabolic rate. *Methods.* Twenty-eight adult women with type 2 diabetes mellitus participated in this cross-sectional study. Anthropometric and biochemical variables were measured as well as body composition (by absorptiometry dual X-ray emission) and basal metabolic rate (by indirect calorimetry); basal metabolic rate was also estimated by prediction equations. *Results.* There was a significant difference between the measured and the estimated basal metabolic rate determined by the FAO/WHO/UNU (*P*_value_ < 0.021) and Huang et al. (*P*_value_ ≤ 0.005) equations. *Conclusion.* The calculations using Owen et al's. equation were the closest to the measured basal metabolic rate.

## 1. Introduction

Type 2 diabetes mellitus (T2DM) is the most common type of diabetes mellitus, characterized mostly by obesity and/or a high abdominal fat percentage [1]. The primary strategy for treatment of obese individuals with type 2 diabetes is the loss of body mass (BM), which is associated with better glycemic control [2].

A precondition for an appropriate dietary prescription with the goal of reducing BM is knowing the daily energy needs of individuals with T2DM, which are determined by the total energy expenditure (TEE) of these individuals. Calculating the TEE of individuals or populations requires knowledge of the basal metabolic rate (BMR), which is the major component of TEE [3].

The BMR is influenced by different factors such as hormonal and body composition changes [4], which are features found in obesity and T2DM. In sedentary individuals, the BMR is about 60 to 70% of TEE [5], and a small change can lead to an energy imbalance and changes in BM [6].

The use of prediction equations is the fastest, simplest, and cheapest way to estimate BMR, taking into consideration factors such as gender, age, mass, height, and LBM. However, several authors have shown that these equations can generate errors that overestimate or underestimate the result, without, however, clarifying the magnitude of these errors [3, 7–9]. These deviations can happen because the characteristics of the population, which we want to evaluate, often differ from the characteristics of individuals who participated in the study which originated these equations [8].

Research conducted in different ethnic groups found that the equations from Harris & Benedict [10], FAO/WHO/UNU [11], Schofield [12], and Henry & Rees [13] overestimated the BMR values particularly in individuals living in tropical countries [13–16]. Wahrlich & Anjos [16] justified these differences by the fact that the equations are derived mostly from samples of American and European populations which show differences in body composition, besides living in different environmental conditions.

In Brazilian women, aged 19–27 years of age, living in Niterói (RJ), the equations from Harris & Benedict [10], FAO/WHO/UNU [11], and Henry & Rees [13] overestimated BMR in 18.9%, 12.5%, and 7.2%, respectively [15]. Similarly, women living in tropical regions, ranging in age from three to 60 years, the equation from Schofield [12] overestimated BMR by 5.4% [13] and by 3.8% in Australian who aged 18 to 30 years [14]. Furthermore, the study from Wahrlich et al. [17], performed with Brazilian residents in the southwestern United States, aged 20–60 years, showed that the equations from Harris & Benedict [10], Schofield [12], and Henry & Rees [13] overestimated the BMR by 8.5% to 15%.

Considering the data presented, it can be inferred that the choice of prediction equations for calculating BMR should be careful. The researchers recommend the use of indirect calorimetry or the development of specific equations for the population of interest or even to validate the prediction equation for the population studied [8, 18].

Several studies have evaluated the BMR of adult women without T2DM using prediction equations [7, 17–20]. However, only a few studies have compared the basal metabolism in adult women with T2DM with the prediction equations [6, 21–23]. Therefore, the aim of the present study is to indicate which of the selected equations most accurately reflects basal metabolism.

## 2. Methods

This cross-sectional study included 92 adult women with T2DM, aged 30–60 years, attended by the Public Health System of Rio de Janeiro (Brazil), from March to December of 2011. Among these, 12 (13.0%) declined to participate. Of the 80 women who accepted the invitation, only 28 (35.0%) were evaluated; 52 were unable to participate because of the following exclusion criteria: 18.8% (*n* = 15) were using insulin; 18.8% (*n* = 15) had thyroid dysfunction; 7.5% (*n* = 6) had cardiomyopathy; 1.2% (*n* = 1) had liver disease; 1.2% (*n* = 1) had nephropathy; and 17.5% (*n* = 14) were smokers.

Informed consent was obtained from all participants in the study. This project was approved by the Ethics Committee in Research from State University of Rio de Janeiro (Brazil) under the number 020.3.2010.

The participants answered a questionnaire to assess personal characteristics, lifestyle habits, physical activity, menstrual cycle, and use of supplements and medication. Sedentary is defined as physical inactivity at the minimum necessary to promote and maintain health (moderate-intensity aerobic physical activity for a minimum of 30 min on five days a week or high-intensity aerobic physical activity for a minimum of 20 min on three days a week).

Anthropometric and body composition evaluations, BMR measurements, and blood samplings were performed at the Interdisciplinary Laboratory of Nutritional Assessment (ILNA) of the Nutrition Institute, State University of Rio de Janeiro (Brazil). The BM (in kilograms) was measured on an electronic platform scale (Filizola; Indústrias Filizola S.A., São Paulo, Brazil) with a maximum capacity of 180 kg and precision of 100 g. Height (in centimeters) was measured using a mobile stadiometer (Alturexata; Alturexata Ltda., MG, Brazil) with an extension of 2 m. Waist circumference (in centimeters) was measured using an inelastic and flexible tape of 0.7 cm width and 0.1 cm accuracy. Measurements were performed by a trained technician with barefoot volunteers wearing minimal clothing and free from accessories, according to the standards of Lohman et al. [24]. The classifications of nutritional status based on body mass index (BMI = kg/m^2^) followed the recommendations of WHO [25].

The body composition assessment to estimate lean body mass (LBM) and fat mass (FM) was performed by the method of dual absorptiometry emission X-ray (DXA; Lunar IDXA, GE, USA; Encore software version 12.2). The BMR was measured by indirect calorimetry using the Vmax Encore 29 Calorimeter System (Viasys Healthcare, Inc., Yorba Linda, CA) calibrated every day before collecting data. The measurement was performed in the morning in a relaxed, temperature-controlled, low-light, and noiseless environment. Following the protocol for measuring BMR, the participants were instructed to arrive at the ILNA by car or public transport upon waking up after sleeping for six to eight hours and fasting for at least 12 hours and avoiding heavy physical exercise and alcoholic intake the day before. Adherence to this protocol was checked before starting the measurement of BMR. After the participants remained initially at rest (supine position) for 20 minutes, the gas exchange was measured for 30 minutes by a canopy. Throughout the test, the participants could not sleep, get up, and/or talk. The volumes of CO_2_ and O_2_ obtained in the first ten minutes were discarded, and the gases obtained during the subsequent 20 minutes of the test were used to determine the BMR in kilocalorie per minute [26]. The means of these values were multiplied by 1440 to obtain the 24-hour BMR. In volunteers who had menstruated, BMR was measured in the follicular phase of the cycle. Two measures of BMR were made in two consecutive days in nine volunteers to assess the quality of the data measured; no variability occurred between the first and second measure (*P*_value_ < 0.05).

The BMR was estimated by seven commonly used prediction equations for women: Harris and Benedict [10], FAO/WHO/UNU [11], Owen et al. [19], Mifflin et al. [20], Gougeon et al. [23], Huang et al. [6], and Rodrigues et al. [18] (Table 1).

Biochemical tests were performed to determine the level of metabolic control; blood samples were collected after an overnight fast of 12 hours on the same day as the BMR measurement. Volunteers were requested to suspend intake of oral hypoglycemic medications on the morning of the biochemical exam day until after blood collection and intake of offered snacks (fruit drink with no added sugar and a whole wheat bread sandwich with no added sugar and white cheese). The following biochemical analyses were performed in the Laboratory of Lipids, Faculty of Medical Sciences, State University of Rio de Janeiro (Brazil): fasting plasma glucose (glucose oxidase/peroxidase method), glycated hemoglobin (immunoturbidimetric assay), total cholesterol (cholesterol oxidase/peroxidase method), HDL-c (direct detergent method), and triglycerides (glycerol phosphate oxidase/peroxidase method). The LDL-c values were calculated by the Friedewald equation, once the plasma triglyceride levels were less than 400 mg/dL. Normal values of biochemical analyses were as follows: fasting plasma glucose of 70–99 mg/dL; glycated hemoglobin < 7.0%; total cholesterol < 200 mg/dL; HDL-c > 50 mg/dL; LDL-c < 100 mg/dL; triglycerides < 150 mg/dL.

The difference between the BMR estimated by equations and BMR measured by indirect calorimetry was calculated (estimated BMR − measured BMR). The percentage of deviation between estimated BMR values for each prediction equation and the measured BMR were calculated as follows: [(estimated BMR − measured BMR)/measured BMR] × 100. The* Kolmogorov-Smirnov* normality test was used to determine the distribution of the variables. Paired Student's *t*-test was used to estimate the statistical significance of the mean difference between the measured and estimated BMR for each prediction equation. The Bland and Altman method [27] was used to evaluate the agreement between the results of the measured and estimated BMR, and Pearson's correlation coefficient was used to assess the correlation between them. The participants were divided into three categories: normal weight, overweight, and obese, according to the classification of nutritional status [25]. The differences between the means of the BM, LBM, FM, fasting plasma glucose, and glycated hemoglobin and the means of BMR (measured, adjusted by BM, and adjusted by LBM) were evaluated by one-way ANOVA in the three categories of nutritional status, followed by Tukey's test. For inferences, a confidence level of 95% was adopted. We used Pearson's correlation between the dependent variable (BMR) and the independent variables of age, height, BM, BMI, waist circumference, FM, LBM, fasting plasma glucose, and glycated hemoglobin.

## 3. Results

The women in the study were all sedentary (*n* = 28); 39.3% (*n* = 11) were of childbearing age; of those on hypoglycemic medication, 71.4% (*n* = 20) used metformin, 7.1% (*n* = 2) used glibenclamide, and 21.4% (*n* = 6) used both. The ages of the volunteers ranged from 37 to 59 years, and BMI results showed that 17.9% (*n* = 5) were normal weight, 17.9% (*n* = 5) were preobese, 32.1% (*n* = 9) were obese class I, 25.0% (*n* = 7) were obese class II, and 7.1% (*n* = 2) were obese class III (Table 2). Total cholesterol and plasma triglyceride levels were within established limits. However, blood glucose, glycated hemoglobin, and LDL-c were elevated, and the HDL-c value was below the normal range, which confirms poor metabolic control. Other biochemical tests were in the normal range (Table 2).

The variables assumed a normal distribution according to the* Kolmogorov-Smirnov test* (data not shown).

Paired Student's *t*-test showed a significant difference between the estimated and measured BMR for FAO/WHO/UNU [11] and Huang et al. [6] prediction equations (Table 3).

According to the deviation percentage, the prediction equation that overestimated the measured BMR the most was that of Huang et al. [6] (11.26%; 4 to 18), followed by FAO/WHO/UNU [11] (10.58%; 3 to 18). Similarly, the equation that underestimated the measured BMR the most was that of Mifflin et al. [20] (−2.58%; −8 to 3), and the equation that estimated most closely the measured BMR was that of Owen et al. [19] The coefficient of variation was 20.62% for measured BMR and 7.51 to 12.56% for estimated BMR (Table 3).

**Figure 1:**
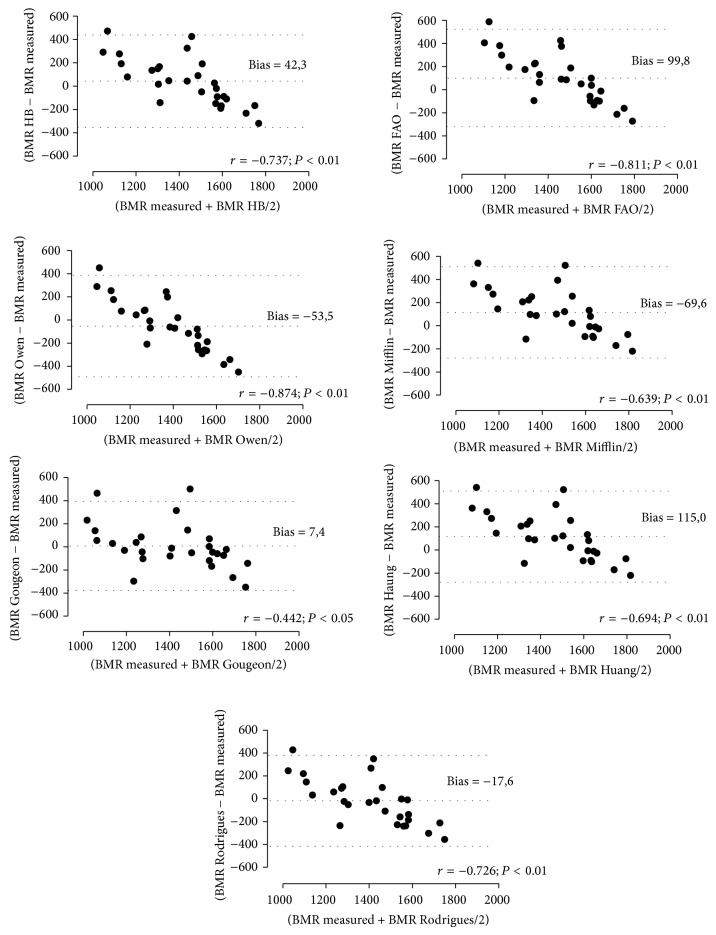
Analysis of Bland and Altman association and the difference between the estimated and measured BMR and the difference between the two methods in women with type 2 diabetes mellitus. BMR: basal metabolism rate; HB: Harris e Benedict; FAO, FAO/WHO/UNU; *r*: Pearson's correlation coefficients.

The graphs demonstrating the agreement between the values of measured and estimated BMR suggest a poor correlation between the two methods, with wide limits of agreement. However, strong negative correlations were observed (*P*_value_ < 0.01) between methods (Figure 1).

In women with diabetes classified as obese, mean BM, LBM, FM, and measured and estimated BMR were significantly higher (*P*_value_ < 0.05) than in women who were nonobese. However, when the BMR was adjusted for BM and LBM, there was no significant difference between groups (Table 4). There was also no significant difference between groups, when we compared the BMR differences and the percentage deviations.

The correlation shows the association between dependent and independent variables, and BMR was significantly correlated (*P*_value_ < 0.01) with BM (*r* = 0.729), BMI (*r* = 0.640), waist circumference (*r* = 0.705), FM (*r* = 0.705), and LBM (*r* = 0.642). There were no significant correlations between BMR and fasting plasma glucose or between BMR and glycated hemoglobin.

## 4. Discussion

There is little research that compares the BMR measured by indirect calorimetry with that estimated by prediction equations in adult women with T2DM. Therefore, we selected five equations developed for healthy adult women with different BM [10, 11, 18–20] but only two equations from populations of obese adults with T2DM [6, 23].

Of the two specific equations for populations with T2DM, the estimations determined by Huang et al. [6] equation were significantly different from the BMR measured by indirect calorimetry in the investigated sample. As for Gougeon et al. [23] equation, there was no significant difference, with overestimation of only 2.80%.

When comparing the prediction equations for the assessment of BMR in adult healthy women [10, 11, 18–20] with the measured BMR in women with T2DM investigated in this study, the results were controversial. Although BMR values were overestimated when determined by Harris and Benedict [10] and Rodrigues et al. [18] equations and underestimated when determined by Mifflin et al. [20] equation, these calculated values were not significantly different from the measured BMR. Only the results determined by the FAO/WHO/UNU [11] equation were significantly different from the measured BMR values. In Ryan et al. [22] study, the FAO/WHO/UNU [11] equation also overestimated the BMR measured in French individuals with T2DM in both genders. However, contradicting the results of this study, the Harris and Benedict [10] equation underestimated the BMR measured in individuals with T2DM in other studies [6, 21]. The Harris and Benedict [10] and FAO/WHO/UNU [11] equations, when used for adult women without diabetes mellitus, tend to overestimate the BMR measured by indirect calorimetry by 5 to 15% [7, 17–20]. The authors of these studies justify this variability by noting that these equations were applied to populations of different racial groups with different body composition and life style. Among the equations selected in this study, that of Owen et al. [19] resulted in values that are the closest to those measured by indirect calorimetry, according to the deviation percentage, with the population of his study being 44 healthy American women, who aged 18 to 65 years, classified as lean to obese.

This study revealed that most of the estimations from the selected equations differed from those measured by indirect calorimetry because the equations cannot estimate values with the same consistency and magnitude as the results determined by gas exchange. Such discrepancies were also observed by Wahrlich et al. [17] among Brazilian women living in the United States. Bland and Altman [27] warned that discrepancies such as these should be interpreted carefully since it may be clinically relevant. Although the current study has revealed poor agreement between the two methods, it is important to emphasize the high negative correlation found between them. However, the correlation indicates only how the two methods are linearly interacted not expressing properly the agreement between them.

There is no scientific evidence indicating how the presence of diabetes mellitus may influence basal metabolism. However, some authors have confirmed higher BMR values in subjects with T2DM compared with controls without the disease [6, 21, 23, 28]. The reason for this increase in BMR is not yet well established, and several mechanisms have been proposed to explain it, such as increased protein turnover [29] and elevated plasma concentrations of free fatty acids in fasting [30] and increased gluconeogenesis in patients with T2DM, which is known to be an energy-consuming metabolic pathway [31]. Consoli et al. [32] observed that the increased gluconeogenesis increases BMR by more than 50% in subjects with T2DM. Another factor to consider is the association between T2DM and excessive body weight, as some authors have shown that obese people have both increased FM and LBM, which contributes to the increased BMR [33, 34].

The LBM, which is the most active metabolic tissue of the body, composed of intra- and extracellular water, proteins, carbohydrates, mineral tissues, and essential lipids [35], is the main determinant of BMR [36]. In the present study, when women with T2DM were classified into three groups according to nutritional status (normal weight, overweight, and obese), measured BMR was significantly lower in normal weight and preobese women than in obese women. However, these differences disappeared when the BMR was adjusted for BM and LBM, confirming the evidence found in the literature that LBM is the main determinant of BMR and indicating also that, for women with T2DM, the BM seems to be a determinant of BMR. However, when we tried to understand if the differences in body composition influenced the estimation error of the selected equations in the study, we found no difference between the BMI groups. Thus, we could not elucidate which of the equations had a lower error between normal weight, overweight, and obese groups.

In women with T2DM evaluated in this study, the best correlation found with BMR was BM (*r* = 0.729). This result is in agreement with the study done with severely obese Australian adults with and without T2DM [6] that found a better correlation (*r* = 0.694) of BM with BMR. These findings corroborate the importance of using the BM as an independent variable in the prediction equations to correctly estimate the BMR of women with T2DM since the equations selected in this study included BM as independent variable [6, 10, 11, 18–20, 23]. It is vital to estimate more accurately the BMR of women with T2DM and preobesity and/or obesity to provide an individualized program for food planning aimed at glycemic and BM control in these patients.

Gougeon et al. [23] evaluated the BMR of women with T2DM, proposing an equation to predict BMR that tested plasma glucose and glycated hemoglobin levels as some of its independent variables, justifying a better adjustment in the model equation. Huang et al. [6] indicated that both the plasma glucose levels and the glycated hemoglobin should be included in the model. However, in this study, although these variables were also considered, there were no significant correlations (*P*_value_ = 0.283 and 0.251) for fasting plasma glucose and glycated hemoglobin, respectively, with BMR. This suggests that other metabolic factors, not controlled in this study, could influence the BMR of women with T2DM.

To obtain a more homogeneous study population and, therefore, observe the characteristics displayed by the evaluated group without the influence of other factors that could affect the basal metabolism, strict inclusion criteria were adopted in this study in the selection of volunteers, which was not always observed in other studies. The rigidity in the selection criteria resulted in a reduction of the sample size, which is one of the limitations of this study. However, it is emphasized that some authors [6, 21, 23], evaluating the BMR of patients with diabetes mellitus, did not take into account the difference between genders, the type of diabetes, or the presence of other diseases.

It is necessary in future research to compare the BMR of individuals with and without T2DM to elucidate the association of T2DM with obesity and other intercurrent factors. Likewise, it is necessary to validate the BMR prediction equations, including women with T2DM with their inherent characteristics, in study populations normally found in public or private clinics.

The findings showed that among the selected prediction equations, the BMR estimated by Owen et al. [19] equation was the closest to the measured BMR as assessed by the percentage deviation.

## Figures and Tables

**Table 1:** Selected prediction equations for estimating basal metabolic rate in women with type 2 diabetes mellitus.

References	Equation for estimation BMR
	(kcal/day)
Harris and Benedict [10]	655.0955 + (9.5634 × BM) + (1.8496 × Ht) − (4.6756 × Age)

FAO/WHO/UNU [11]	(8.7 × BM) + 829

Owen et al. [19]	795 + (7.18 × BM)

Mifflin et al. [20]	(10 × BM) + (6.25 × Ht) − (5 × Age) − 161

Gougeon et al. [23]	375 + (85 × BM) − (48 × FM) + (63 × FPG)

Huang et al. [6]	71.767 − (2.337 × Age) + (257.293 × 0) + (9.996 × BM) + (4.132 × Ht) + (145.959 × 1)

Rodrigues et al. [18]	BMI > 35 kg/m^2^: 172.19 + (10.93 × BM) + (3.10 × Ht) − (2.55 × Age)
	BMI < 35 kg/m^2^: 407.57 + (9.58 × BM) + (2.05 × Ht) − (1.74 × Age)

BM: body mass (kg); Ht: height (cm); FPG: fasting plasma glucose (mM)^*^; BMI: body mass index (kg/m^2^).

^*^Unit of measure described in the original article of the equation.

**Table 2:** Characteristics of women with type 2 diabetes mellitus.

Variable	Mean	95% CI
Age (year)	51	(49; 54)
Body mass (kg)	78.4	(72.9; 84.0)
Height (cm)	156.0	(153.8; 158.3)
BMI (kg/m^2^)	32.1	(30.0; 34.2)
WC (cm)	95.7	(91.6; 99.8)
LBM (kg)	45.0	(42.5; 47.5)
FM (kg)	33.0	(29.6; 36.3)
FPG (mg/dL)	141.6	(117.4; 165.8)
A1C (%)	7.7	(6.7; 8.6)
Total cholesterol (mg/dL)	195.3	(181.0; 209.6)
HDL-c (mg/dL)	48.7	(45.5; 51.9)
LDL-c (mg/dL)	119.0	(106.1; 132.0)
Triglycerides (mg/dL)	137.6	(111.3; 163.9)

95% CI: 95% confidence interval; BMI: body mass index; WC: waist circumference; LBM: lean body mass; FM: fat mass; FPG: fasting plasma glucose; A1C: glycated hemoglobin.

**Table 3:** Comparison between the estimated and measured BMR in women with type 2 diabetes mellitus.

Variable	Mean	(95% CI)	*P*_value_
Measured BMR (kcal in 24 h)	1411.9	(1300.2; 1523.2)	
Estimated BMR (kcal in 24 h)			
Harris and Benedict [10]	1453.9	(1397.8; 1511.1)	
Difference^ (1)^ (kcal in 24 h)	42.3	(−36.1; 120.6)	0.278
Deviation %^ (2)^	5.9	(−0.7; 12.6)	
FAO/WHO/UNU [11]	1511.5	(1463.5; 1559.4)	
Difference^ (1)^ (kcal in 24 h)	99.8	(16.5; 183.1)	0.021^*^
Deviation %^ (2)^	10.6	(2.9; 18.2)	
Owen et al. [19]	1358.2	(1318.6; 1397.8)	
Difference^ (1)^ (kcal in 24 h)	−53.5	(−140.5; 33.5)	0.218
Deviation %^ (2)^	−0.5	(−7.5; 6.5)	
Mifflin et al. [20]	1342.1	(1276.7; 1407.4)	
Difference^ (1)^ (kcal in 24 h)	−69.6	(−146.6; 7.4)	0.075
Deviation %^ (2)^	−2.6	(−8.4; 3.2)	
Gougeon et al. [23]	1419.1	(1339.1; 1499.0)	
Difference^ (1)^ (kcal in 24 h)	7.4	(−69.2; 83.9)	0.845
Deviation %^ (2)^	2.8	(−3.7; 9.3)	
Huang et al. [6]	1526.7	(1466.0; 1587.1)	
Difference^ (1)^ (kcal in 24 h)	115.0	(36.9; 193.1)	0.005^*^
Deviation %^ (2)^	11.3	(4.2; 18.4)	
Rodrigues et al. [18]	1394.0	(1336.3; 1451.8)	
Difference^ (1)^ (kcal in 24 h)	−17.6	(−96.4; 61.1)	0.649
Deviation %^ (2)^	1.5	(−4.9; 8.0)	

Paired Student's *t*-test: ^*^ *P*_value_ < 0.05. BMR: basal metabolic rate; 95% CI: 95% confidence interval.

^(1)^(Estimated − measured) (kcal in 24 h).

^(2)^(Difference/measured) × 100 (%).

**Table 4:** Difference between the means of anthropometric, biochemical, and body composition of women with diabetes mellitus type 2 classified according to nutritional status [18].

	Normal weight		Pre-obese		Obese	
	(*n* = 5)		(*n* = 5)		(*n* = 18)	
	Mean	(95% CI)	Mean	( 95% CI)	Mean	( 95% CI)
Age (years)	52.6	(43.2; 62.0)	52.2	(43.0; 61.4)	50.7	(47.6; 53.9)
BM (kg)	57.0	(52.0; 62.0)^ab^	67.5	(62.2; 72.9)^ac^	87.4	(83.7; 91.2)^bc^
LBM (kg)	36.8	(34.9; 38.8)^b^	39.5	(35.9; 43.1)^c^	48.8	(46.7; 50.9)^bc^
FM (kg)	20.0	(14.2; 25.8)^ab^	27.8	(23.4; 32.2)^ac^	38.0	(35.6; 40.5)^bc^
FPG (mg/dL)	129.0	(67.2; 190.8)	102.8	(95.7; 109.9)	155.9	(121.1; 190.7)
A1C (%)	7.4	(5.6; 9.2)	6.2	(5.5; 7.0)	8.1	(6.7; 9.5)
BMR (kcal)	1129.2	(905.0; 1352.4)^b^	1211.8	(1044.9; 1378.7)^c^	1545.7	(1418.7; 1672.7)^bc^
BMR/BM (kcal*·*kg^−1^)	19.9	(15.2; 24.6)	18.0	(15.7; 20.2)	17.7	(16.4; 18.9)
BMR/LBM (kcal*·*kg^−1^)	30.7	(24.3; 37.1)	30.6	(28.4; 32.8)	31.8	(29.1; 34.4)

**Estimated BMR (kcal)**						
Harris and Benedict [10]	1239.9	(1165.5; 1314.2)^b^	1342.5	(1274.3; 1410.7)^c^	1544.3	(1502.3; 1586.4)^bc^
Difference^ (1)^	110.7	(−90.2; 311.5)	130.7	(2.3; 259.1)	−1.3	(−133.0; 110.4)
Deviation %^ (2)^	11.7	(−7.7; 31.1)	11.7	(−1.4; 24.8)	2.7	(−6.7; 12.1)
FAO/WHO/UNU [11]	1324.6	(1281.1; 1368.0)^ab^	1416.6	(1369.9; 1463.2)^ac^	1589.7	(1557.2; 1622.2)^bc^
Difference^ (1)^	195.4	(−36.6; 427.3)	204.8	(55.9; 353.7)	44.1	(−69.9; 158.0)
Deviation %^ (2)^	19.7	(−4.0; 43.5)	18.0	(2.0; 34.1)	6.0	(−4.3; 16.3)
Owen et al. [19]	1203.9	(1168.1; 1239.9)^ab^	1279.9	(1241.5; 1318.4)^ac^	1422.8	(1396.0; 1449.6)^bc^
Difference^ (1)^	74.8	(−155.3; 304.8)	68.1	(−83.0; 219.3)	−122.9	(−238.5; −7.2)
Deviation %^ (2)^	8.8	(−12.7; 30.3)	6.7	(−8.0; 21.3)	−5.1	(−14.4; 4.2)
Mifflin et al. [20]	1111.9	(1010.3; 1213.6)^b^	1218.4	(1120.7; 1316.1)^c^	1440.3	(1386.4; 1494.3)^bc^
Difference^ (1)^	−17.2	(−228.9; 194.5)	6.6	(−115.6; 128.9)	−105.3	(−216.2; 5.5)
Deviation %^ (2)^	0.1	(−17.6; 17.8)	1.2	(−9.3; 11.7)	−4.4	(−12.8; 4.0)
Gougeon et al. [23]	1126.5	(1078.5; 1174.5)^b^	1235.2	(1149.3; 1321.2)^c^	1551.4	(1495.9; 1606.8)^bc^
Difference^ (1)^	−2.7	(−240.2; 234.9)	23.4	(−97.5; 144.4)	5.7	(−106.5; 117.9)
Deviation %^ (2)^	1.9	(−19.1; 22.9)	2.6	(−8.0; 13.1)	3.1	(−6.3; 12.5)
Huang et al. [6]	1303.1	(1224.2; 1381.9)^b^	1408.8	(1333.1; 1484.6)^c^	1621.5	(1575.1; 1667.8)^bc^
Difference^ (1)^	173.9	(−50.5; 398.2)	197.1	(67.8; 326.3)	75.8	(−34.9; 186.5)
Deviation %^ (2)^	17.6	(−4.6; 39.8)	17.2	(3.5; 30.9)	7.8	(−2.01; 17.7)
Rodrigues et al. [18]	1178.8	(1116.3; 1241.3)^b^	1280.4	(1219.4; 1341.4)^c^	1485.4	(1441.3; 1529.4)^bc^
Difference^ (1)^	49.6	(−173.4; 272.6)	68.6	(−66.7; 203.9)	−60.3	(−171.0; 50.5)
Deviation %^ (2)^	6.4	(−13.7; 26.6)	6.6	(−6.3; 19.4)	−1.2	(−10.2; 7.8)

BM: body mass; LBM: lean body mass; FM: fat mass; FPG: fasting plasma glucose; A1C: glycated hemoglobin; BMR: basal metabolic rate; BMR/BM: basal metabolic rate adjusted for BM; BMR/LBM: basal metabolic rate adjusted for LBM; 95% CI: 95% confidence interval.

One-way ANOVA-Tukey: ^a  b  c^same letters express significant difference between groups.

^(1)^(Estimated − measured) (kcal in 24 h).

^(2)^(Difference/measured) × 100 (%).

## Abbreviations

T2DM: Type 2 diabetes mellitus
BM: Body mass
TEE: Total energy expenditure
BMR: Basal metabolic rate
ILNA: Interdisciplinary Laboratory of Nutritional Assessment
BMI: Body mass index
LBM: Lean body mass
FM: Fat mass
DXA: Dual absorptiometry emission X-ray
$\beta_{\text{value}}$: Regression coefficients
$R^{2}$: Coefficient of determination
Ht: Height
FPG: Fasting plasma glucose
95% CI: 95% confidence interval
WC: Waist circumference
A1C: Glycated hemoglobin
BMR/BM: Basal metabolic rate adjusted for BM
BMR/LBM: Basal metabolic rate adjusted for LBM
HB: Harris-Benedict equation
FAO/WHO/UNU: Food and Agriculture Organization/World Health Organization/United Nations University equation
r: Pearson’s correlation coefficients.
